# Next-Generation Sequencing in Newborn Screening: A Review of Current State

**DOI:** 10.3389/fgene.2021.662254

**Published:** 2021-05-26

**Authors:** Ziga I. Remec, Katarina Trebusak Podkrajsek, Barbka Repic Lampret, Jernej Kovac, Urh Groselj, Tine Tesovnik, Tadej Battelino, Marusa Debeljak

**Affiliations:** ^1^Clinical Institute for Special Laboratory Diagnostics, University Children’s Hospital, University Medical Centre Ljubljana, Ljubljana, Slovenia; ^2^Faculty of Medicine, Institute of Biochemistry and Molecular Genetics, University of Ljubljana, Ljubljana, Slovenia; ^3^Department of Endocrinology, Diabetes and Metabolic Diseases, University Children’s Hospital, University Medical Centre Ljubljana, Ljubljana, Slovenia; ^4^Chair of Pediatrics, Faculty of Medicine, University of Ljubljana, Ljubljana, Slovenia

**Keywords:** newborn screening, NBS, neonatal screening, next generation sequencing, NGS, expanded NBS program, DNA sequencing, high-throughput sequencing

## Abstract

Newborn screening was first introduced at the beginning of the 1960s with the successful implementation of the first phenylketonuria screening programs. Early expansion of the included disorders was slow because each additional disorder screened required a separate test. Subsequently, the technological advancements of biochemical methodology enabled the scaling-up of newborn screening, most notably with the implementation of tandem mass spectrometry. In recent years, we have witnessed a remarkable progression of high-throughput sequencing technologies, which has resulted in a continuous decrease of both cost and time required for genetic analysis. This has enabled more widespread use of the massive multiparallel sequencing. Genomic sequencing is now frequently used in clinical applications, and its implementation in newborn screening has been intensively advocated. The expansion of newborn screening has raised many clinical, ethical, legal, psychological, sociological, and technological concerns over time. This review provides an overview of the current state of next-generation sequencing regarding newborn screening including current recommendations and potential challenges for the use of such technologies in newborn screening.

## Introduction

Newborn screening (NBS) began with the invention by Dr. Robert Guthrie of a relatively simple and rapid test for the detection of elevated levels of phenylalanine in the blood, combined with an ingenious method of sampling, which later proved to be useful in a multitude of different tests (Guthrie, 1961). The success of screening for phenylketonuria (PKU) spurred the expansion of the NBS program (Guthrie and Whitney, 1965; Brosco and Paul, 2013; Groselj et al., 2014a). In the 1970s, the screening began for congenital hypothyroidism (CH), and in the next two decades, a few other disorders like congenital adrenal hyperplasia (CAH), hemoglobinopathies, biotinidase deficiency, cystic fibrosis (CF), and tyrosinemia type I (HT1) were added sporadically to the different NBS programs in different states and countries (Dussault et al., 1975; Wilcken and Wiley, 2015). With the development and the accessibility of electrospray ionization (ESI) tandem mass spectrometry (TMS) in the 1990s, the ability to quantitate multiple metabolites simultaneously and, thus, the simultaneous detection of multiple inborn errors of metabolism (IEM) facilitated the first big expansion of the NBS programs around the globe, though the less developed countries did generally not share the progress (Sweetman, 1996; Levy, 1998; Bennett and Rinaldo, 2001; Groselj et al., 2014a,b; Bouvier and Giguère, 2019). Different tests are continuously developed spurred by the development of clinical treatments for conditions such as severe combined immunodeficiencies (SCID), spinal muscular dystrophy (SMA), and lysosomal storage diseases (Spacil et al., 2013; Chien et al., 2017; Puck, 2019; Czibere et al., 2020). With the constant growth of conditions added to the NBS programs, the number of different analytical methods used is also increasing. It soon became apparent that the use of different methods results in a much heavier workload for the laboratories. At the same time, the progress of genetics was accelerated with the development of next-generation sequencing (NGS) methods (Metzker, 2010; Morey et al., 2013; Reuter et al., 2015; Levy and Myers, 2016). NGS is an epitome of multiparallel platforms because it enables simultaneous processing of a large number of samples, and it is easily expandable from a couple of genes to a whole genome. Many authors therefore see it as the method that could enable the next big expansion and methodological unification of NBS programs (Department of Health, 2003; Collins, 2009; Drmanac, 2012; Smon et al., 2018; Lampret et al., 2020). As the cost of sequencing steadily decreases, the feasibility of NBS with the use of NGS is becoming more and more possible (Wetterstrand, 2021).

**FIGURE 1:**
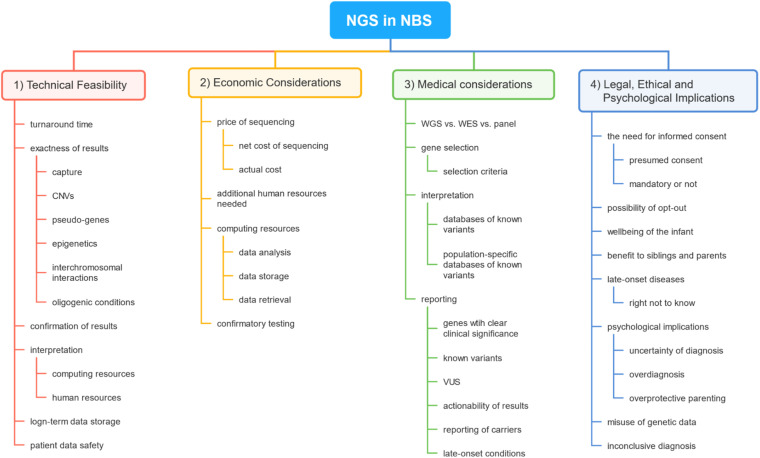
A brief outline of topics covered in this review. The first section covers concerns regarding the basic technical feasibility of the use of next-generation sequencing (NGS) technology in newborn screening (NBS). The second section reveals the main factors impacting the cost of the use of such technology in NBS. The third section deals with medical problems the use of NGS would raise in NBS. The final sections engage in discussing legal, ethical, and psychological problems concerning NGS in the setting of NBS.

However, despite growing technical possibilities, most of the human disorders are not suitable to be included in the NBS program. Wilson and Jungner proposed 10 criteria that should be met for the disease to be included in screening programs, which were later further revised (Wilson et al., 1968; Andermann, 2008). Since the first idea of using NGS in the framework of NBS, several concerns have been raised, among them technical, medical, legal, economical, ethical, psychological, and sociological (Friedman, 2015; Howard et al., 2015; Reinstein, 2015; Berg et al., 2017; Murray et al., 2018; Bouvier and Giguère, 2019; de Wert et al., 2020). In this review, we will discuss the recent advances in the use of NGS in the context of NBS, the remaining obstacles in its implementation in NBS, and the wider implications of its use in the NBS program. A brief outline of this review is demonstrated in Figure 1.

## Technical Feasibility

Since the first idea of using genetic methods in the NBS setting, numerous authors presented several concerns regarding the technical feasibility of using genomic technologies for NBS: possibility to achieve appropriate turnaround time, accuracy of the obtained results, ability to correctly interpret the results, confirmation of the results with an independent method, and safe storage of sequenced data (Friedman, 2015; Howard et al., 2015; Reinstein, 2015; Berg et al., 2017; Bouvier and Giguère, 2019).

The first technical concern is the ability to prepare, analyze, interpret, and report the results fast enough to fulfill the requirements of the NBS. Standard protocols for NGS library preparation are time-consuming and could not be used in an NBS time frame. The second time-consuming step of the NGS approach is the interpretation of the results. One of the largest obstacles would be the interpretation of the sheer bulk of data containing many variants for which their clinical significance is still unknown or ambiguous (Berg et al., 2017). There is still not enough ethnic-specific data available for an accurate evaluation of the possible causality of many such variants (Friedman, 2015). However, there are new optimized protocols reported, enabling the possibility of the results to be reported on day 4 from sample booking in the laboratory (van Campen et al., 2019).

Different NGS approaches yield different technical problems regarding raw sequencing data. Roughly, there are three main approaches to NGS: sequencing of the panels of selected genes, sequencing of coding regions of all known genes or whole-exome sequencing (WES), and sequencing of the whole genome (WGS). When using WES or other targeted approaches (panels of genes), we have to capture the selected regions of DNA and then use enrichment of them for further processing. Here, we risk that we would fail to capture certain parts of specific targeted genes, which could lead to false-negative results. With WGS, there is no need for capture or enrichment, so there is less possibility for certain regions to have poor sequencing coverage (Reinstein, 2015). WGS enables better detection of the copy number variants (CNV), which could be missed using a targeted approach. Some software and technical solutions have already been developed, to address this problem in targeted methods (Belkadi et al., 2015; Kerkhof et al., 2017; Mu et al., 2019). Often overlooked is also the problem of sequencing of genes with known pseudogenes with a high level of homology, which could result in false results of sequencing in all the abovementioned methods, including WGS (García-García et al., 2016). Performing NBS with WGS would also require numerous high-throughput next-generation sequencers (including backup machines) to facilitate uninterrupted NBS, which still have a very high price, which is possibly beyond many countries’ healthcare systems capacity (Schwarze et al., 2020).

Regardless of the approach, WGS or targeted, there are some properties of the human genome being uncovered, that neither could be detected without complex technical modifications or a special type of human genome analysis. The role of epigenetics and non-coding RNAs on the expression of genes is just starting to be discovered (Tesovnik et al., 2020). The role of topologically associated domains and interchromosomal interactions and the influence of the 3D structure of the genome in the expression of genes and potential cause of the disease are still not fully understood (Maass et al., 2018, 2019). Oligogenic or multifactorial conditions are only starting to be understood, but could in the future be a very important part of preemptive medicine. Presently, it is difficult to predict which kind of methodological approach would be better for the detection of oligogenic conditions, but it seems that WGS would be more appropriate (Tada et al., 2018; Kim et al., 2019). As it seems that all of the abovementioned conditions and diagnostic methods have the potential, with the maturation of the analytical technology and increasing knowledge of their impact on human health, they become part of the NBS in the future.

Sequencing of the whole population would produce an enormous amount of computer data that would have to be stored safely for a long time depending on the local regulation of medical data storage, handling, and distribution. Some authors propose that newborns would be screened/sequenced once in a lifetime, then their genomic data would have to be stored securely for an entire lifespan or even more. In such cases, data would have to be accessible for subsequent reinterpretation as new knowledge regarding genes and related diseases becomes available (Drmanac, 2012; Friedman, 2015; Howard et al., 2015).

Integration of obtained data and results is another challenge for informatics technologies. Because of the sensitivity of genetic data, permissions for access to the results and the ability to interpret the data would have to be thoughtfully assigned. Currently, the task of interpreting genetic data is exclusively in the domain of clinical and laboratory geneticists (CLGs), which is a well-recognized profession worldwide. Some authors propose that some part of the increased analytical burden in NBS could in the future be taken over by medical doctors (MDs) (Friedman, 2015; Howard et al., 2015; Berg et al., 2017). If we consider the part MDs play in healthcare systems and their assignments, we usually conclude that they are already overburdened. Although it is conceivable that MDs could interpret some results of the simpler genetic tests, the majority of analytical burden will and should remain on specialists of clinical and laboratory genetics (Liehr et al., 2017, 2019).

Regardless of the abovementioned technical hurdles, NGS simply could not provide a unified platform for NBS, as some authors suggest, because some of the diseases presently screened have a very weak genetic background or very variable penetrance. A couple of such examples are CH for which only 10–15% of patients have a genetic background (Rastogi and LaFranchi, 2010), some conditions of fatty-acid metabolism, and Pompe disease, which does not have a clear genotype–phenotype correlation (Matern and Rinaldo, 1993; Kroos et al., 2012). Another obstacle of using NGS as a first-tier method in some disorders is the sensitivity of sequencing analysis, especially when analyzing a selected panel of known genetic variants such as in cystic fibrosis (Castellani and Massie, 2014). For many of the known genetic diseases, there is currently no valid clinical evaluation by which to validate genomic prediction (Berg et al., 2017).

## Economic Considerations

With the advancement of NGS methods, the price of sequencing per one million bases (1 Mb) dropped significantly and continues to drop even more (Wetterstrand). Nonetheless, the aspirational price of the whole human genome sequencing is still advertised around a $1,000 mark, but depending on the country and equipment used, it can be increased by multiple factors including the costs of accreditation, validation, maintenance, and external quality control, and specialists of different fields (genetics, bioinformatics, etc.) can raise the cost of a single human genome much higher (Liehr, 2017; Schwarze et al., 2020). With or without additional costs, the cost of NGS is still significantly higher than the current cost per sample in all of the existing NBS programs. For example, the reported cost per newborn in Europe ranged from €1 (Moldova; screening for 2 conditions) to €43.24 (the Netherlands; screening for 17 conditions) (Groselj et al., 2014b; Loeber et al., 2021). In the United States, most states fund the NBS program through a “kit fee” which is paid to the birthing facility which is currently roughly $100 per newborn to screen for over 30 conditions (Botkin and Rothwell, 2016). In Israel, they screen for 12 diseases and the cost per sample is estimated around $45 (Friedman, 2015).

Another important aspect of cost is the computing and human power required to process the data obtained with NGS. After data are processed, there would be the need to interpret the clinical significance of obtained variants. With the increase in the number of sequenced genes, there would also be an increase in the number of variants found that would need interpretation. Many software tools are already available to ease the process of interpretation of results, but in the end, there is still the need for highly trained geneticists to make a final decision. With these much data, interpretation would require a great increase in manpower compared with the current workflow (Howard et al., 2015). To address this problem, some authors propose automated software pipelines that would report only on currently known pathogenic and likely pathogenic variants (Berg et al., 2017; van Campen et al., 2019).

The need to confirm the results obtained through genetic screening would possibly increase the cost of confirmatory testing because of the greater number of screened conditions. There are several possibilities of confirming NGS results. For known genes, the golden standard is still Sanger sequencing of the variant in question. If we consider WGS, the use of the so-called “trio” approach, comparing WGS results of a child to the results of parents, could be useful for diagnosing *de novo* autosomal dominant intellectual disabilities and autosomal recessive disorders, but the price, time frame, and workload will be beyond the scope of NBS for some considerable time (Mu et al., 2016; Rossi et al., 2020). Genetic NBS would, depending on the design, possibly need informed consent (parental permission) that would require counseling before testing and again when returning the results, which would again increase the need for CLGs’ time and, therefore, require more manpower (Frank et al., 2013). As it was always the case in NBS, every screening for a new condition invariably increases the workload in the clinic where patients get treated.

The quantity of data produced by NGS can be enormous. The size of a human genome on a computer disk represents around 60 gigabytes (GB), which would significantly increase data storage requirements. Long-term digital data persistence and safety would have to be guaranteed. The average lifespan of data on a hard drive is currently between 5 and 6 years, which does not provide long-term data storage, so other solutions would have to be developed (Rahmanto and Riasetiawan, 2018). Many authors predict that genetic data obtained at NBS would be useful throughout the person’s life, especially when considering WGS, where a lot of information is yet to be uncovered.

## Clinical Issues

The current debate regarding the use of NGS in the setting of NBS revolves around the question which sequencing approach is better, WGS or targeted. The authors advocating the WGS approach claim that having the information of the whole genome at the clinician’s disposal would enable not only NBS analysis but further analysis of the genes and regulatory regions of DNA later in a patient’s life when further knowledge on causative variants and genome regions would be available. The authors also claim that the study of data acquired during NBS would enable research of gene variants on a population scale, which would have a substantial benefit on public healthcare in the near future (Drmanac, 2012; Howard et al., 2015; Reinstein, 2015). Another emerging field that would benefit greatly from the data obtained with WGS is personalized medicine, namely pharmacogenomic, which could use genomic data from NBS later in life, to determine patients’ specific drug-metabolizing traits. According to the working group of the personalized laboratory medicine of the European Federation of Laboratory Medicine, there are some recognized organizational shortcomings that impede the progress of pharmacogenomics, among them technological and methodological deficits (Malentacchi et al., 2015; Karas Kuželički et al., 2019). WGS also enables research of non-coding regions that we know can harbor deleterious variants (Meienberg et al., 2016) but are more challenging to properly interpret. Another option would be WGS and analysis of only selected regions/genes. Such an approach would facilitate easier expansion of core NBS diseases and interpretation of genes for new conditions (Di Resta et al., 2018).

Whole-exome sequencing, on the other hand, covers exclusively the coding regions of most of the known genes and is therefore only 1.5% the size of the whole genome, which means faster and cheaper sequencing, but still with a relatively large amount of genomic data. A more “traditional” approach would be a gradual implementation of NGS technology, and several authors suggest a selection of genes for which gene–disease correlation is well known (Evans et al., 2013; Howard et al., 2015; Berg et al., 2017). NGS with a targeted approach, with the use of capture probes for selected panels of genes or with the use of software filters in the case of WGS, has proven its utility in routine diagnostics, so many laboratories already have experience with such methods (Di Resta et al., 2018). The targeted approach would adopt the same inclusion process as was used until now for biochemical screening but now focused on the NGS methodology. Conditions would be added gradually after careful consideration. One important advantage of the targeted NGS approach is its scalability. Conditions can be added with little or no changes or additional cost to the established workflow at all. With this approach, the interpretation of the variants would be less time-consuming and would better fit into the strict NBD time frame, while at the same time the whole process would allow a much greater level of automation, including bioinformatics analysis.

### Selection of Gene–Disease Pairs

Considering the targeted approach, the main challenge would be the selection process of the included genes associated with the targeted disease. Several documents relevant to the use of genetic technologies in NBS have been published over the years. Some of them were written in the field of NBS and others written in the field of medical genetics. Because of the lack of specific guidelines for the use of NGS in NBS, the use of a combination of different documents would be required. At the same time, an expert panel to establish specific guidelines and recommendations for the introduction of NGS as a first-tier screening technology is well overdue, although some international projects are aiming to fill that void in the next couple of years.

The oldest document for screening programs is the WHO criteria, which would have to be met and at the same time considered from an NGS point of view (Wilson et al., 1968; Andermann, 2008). The suitable candidate conditions would have to have a clear Mendelian inheritance pattern and clear genotype–phenotype correlation. There should be ample knowledge of known variants present in the gene, high penetrance, and effective presymptomatic intervention (Howard et al., 2015; Berg et al., 2017; Bouvier and Giguère, 2019). If we consider the current set of core conditions included in NBS programs, some of them do not meet the above criteria, so could not be included in NGS screening, but would have to be screened with existing methods (Green et al., 2006; Rastogi and LaFranchi, 2010; Howard et al., 2015).

In the United States recently, the Discretionary Advisory Committee on Heritable Disorders in Newborns and Children updated the panel of recommended disorders for NBS. The RUSP includes 57 conditions including 31 core disorders and 26 secondary disorders (Health Resources and Services Administration, 2020). The authors suggest that this panel could serve as a template for the selection process of conditions that would be screened using NGS (Evans et al., 2013; Berg et al., 2017; Di Resta et al., 2018; Bouvier and Giguère, 2019). Others argue that it would be difficult to acquire the same specificity and sensitivity for conditions currently screened with the TMS method (Castellani and Massie, 2014; Howard et al., 2015). That is why some propose to include only the disorders for which currently there is no valid method for screening (Berg et al., 2017). The American College of Medical Genetics and Genomics (ACMG) issued recommendations for reporting of incidental findings in clinical exome and genome sequencing in 2013. They list several conditions and genes for which they conclude that known pathological variants in those genes should be reported (Green et al., 2013). Evans et al. (2013) propose only a few of the genes from the ACMG list, for example, genes associated with Lynch syndrome, some highly penetrant cancer predisposition genes (e.g., *APC*, *BRCA1*, *BRCA2*, *MYH*, *PTEN*, and *VHL*), genes associated with high risk for preventable vascular catastrophe (e.g., *FBN1*, *COL3A1*, and *MYH11*), and possibly genes for familial hypercholesterolemia (Evans et al., 2013; Klančar et al., 2015; Groselj et al., 2018). The European Society of Human Genetics (ESHG) published recommendations on opportunistic genomic screening, where for minors they approve reporting of variants only for genes of conditions that are actionable early in life (such as MEN type 2A and hereditary arrhythmias such as long QT and Brugada syndrome) (de Wert et al., 2020). Recently, Milko et al. (2019) published a novel method to assess the potential actionability of genomic sequencing for certain conditions based on age. Such semiquantitative methods could pave the way to a more evidence-based and uniform decision-making process regarding the inclusion of disorders and related genes into an NGS-based NBS program (Berg et al., 2016; Strande et al., 2017).

### Interpretation and Reporting of Genetic Variants

Many authors expressed concerns regarding the clinical interpretation of acquired data. In WGS/WES, there is an enormous bulk of obtained information that is far from easy to analyze even with powerful computing power. If we disregard the time it takes to process the data, there is still the question of reliable analysis and interpretation of obtained data. Depending on the software and databases used, interpretation will inevitably vary between laboratories (Levy, 2014). The cause of this variation is the current lack of knowledge about genetic variants, many of which are presently classified as variants of unknown significance (VUS) (Cooper and Shendure, 2011; O’Daniel et al., 2017). New national databases of variants should be developed, to decrease VUS and improve population-specific knowledge on variants. Besides incomplete knowledge about genetic variants, classification of the same variant with different databases or predictive algorithms is often conflicting, which would make reliable interpretation even more difficult. With the inception of genomic NBS, many more variants of unknown significance will be uncovered because of the sheer volume of samples sequenced (Lyon and Wang, 2012; Rabbani et al., 2012).

With the genome or at least parts of it at our disposal, it remains to be determined which variants would be included in the report and returned to the parents in the end. Considering information obtained with WGS, the data will contain many very different types of genetic variants, from known variants in genes for monogenic conditions to polygenic conditions, childhood-onset to late-onset disorders, and autosomal dominant or recessive (homozygous or carrier) to X-linked or mitochondrial; variants for certain predispositions with variable penetrance and pharmacogenomic data; and last but not least, VUS (Wade et al., 2013; Malentacchi et al., 2015). In the case of WGS or WES, we must take into account that the acquired information will include all of the genes even the ones whose clinical significance is not yet understood. Some sort of scrutiny regarding actionability and gene–disease association will be required when reporting genomic or exomic data (Friedman, 2015). In most of the current conditions included in the NBS, the heterozygous carriers never develop clinical signs or symptoms, and in very few actionable conditions, heterozygous carriers develop them already in childhood. This raises the question how and when, if at all, should this information be reported. We learned from the experience of the NBS program for sickle cell disease that reporting of genetic carriers can do more harm than good (Rutkow and Lipton, 1974; Tarini and Goldenberg, 2012). On the other hand, in Fabry disease, some heterozygous females can develop full clinical presentation and should be identified to issue enzyme replacement treatment (Ortiz et al., 2018).

There is also the dilemma of late-onset conditions for which reporting to asymptomatic minors has been, until recently, only recommended when established prevention or treatment that may alter the course of the condition is available at the time of testing (Borry et al., 2009; van El et al., 2013; Howard et al., 2015; Bondio, 2017). A revised version of ACMG recommendations for reporting of secondary findings in clinical exome and genome sequencing is the first document that recommends reporting of information on select few actionable late-onset conditions in minors, with the explanation that this information could prove invaluable for the health of the parents (Kalia et al., 2017). The ESHG published recommendations for opportunistic genomic screening in which they do not object to reporting of population pharmacogenomics (PGx) variants and variants leading to early onset actionable conditions (de Wert et al., 2020). Despite professional guidelines deferring asymptomatic testing, carrier testing, or testing for late-onset diseases in minors, the public opinion may be in favor, as studies suggest (Shkedi-Rafid et al., 2015; Waisbren et al., 2015).

## The Need for Informed Consent

Several current NBS programs are mandatory with most of them offering an opt-out option (Tarini and Goldenberg, 2012; Lampret et al., 2020; Loeber et al., 2021). Many of them are free of charge and do not require parental permission because in most NBS programs the participation is still considered in the best interest of the child’s health. The model of presumed consent is still considered to be the most suitable in the context of NBS for conditions for which clear benefit to the child has been proven (Howard et al., 2015). When we consider some form of genomic screening, the consent and counseling of parents seem requisite. Currently, all genetic testing requires informed consent to be obtained from the patient, parents, or legal guardian during a genetic counseling session performed by a medical geneticist or similar, before the testing (ACMG Board of Directors, 2013). Many authors argue that if NBS will be based on genetic testing, some form of informed consent will be necessary (Friedman, 2015; Howard et al., 2015; Berg et al., 2017; de Wert et al., 2020). Others propose solutions such as a panel of core conditions with proven benefits for the child for which no consent would be necessary (Tarini and Goldenberg, 2012; Friedman, 2015). One study researched the possibility of using an electronic decision aid to assist parents in decision-making regarding genetic NBS which would guide them to informed consent they would understand (Berg et al., 2017). A multistep or dynamic informed consent has been proposed in case of reporting secondary findings in cancer patients (Pujol et al., 2018). Some authors fear that the need for explicit consent could lead to reduced participation in the NBS program and would pose an additional burden to healthcare workers involved (Jepson et al., 2001; Feuchtbaum et al., 2007; Tarini and Goldenberg, 2012).

## Ethical and Psychological Implications

The WHO principles for screening programs have been the center of every NBS program. Emanating from those principles, the focus of NBS has always been on the well-being of the infant to the degree that in many countries the opportunity to intervene and dramatically improve an infant’s course of life supersedes parental autonomy (Wilson et al., 1968; Tarini and Goldenberg, 2012; Howard et al., 2015).

With the prospect of genetic testing of newborns, various authors see several different benefits of such testing, not pertaining solely to the child (Bombard et al., 2010; Drmanac, 2012; Best et al., 2018; Lantos, 2019). The ACMG proposal recommends the reporting of variants in the selected 59 highly penetrant and actionable genes, no matter what the indication for which clinical sequencing was requested and regardless of patient’s age. The rationalization for such recommendation is that for these genes the benefit of other family members outweighs the child’s right not to know. The proposed benefits of testing a minor or in the case of NBS a newborn for an adult-onset disease would be a benefit of the parent or sibling who could become ill with the disease in question and could benefit from the NBS results of the child. The result could also enable parents to make informed decisions about future pregnancies (McGuire et al., 2013; Wilfond et al., 2015; de Wert et al., 2020). Reporting of such variants would inevitably put enormous strain on the health system, with entire families requiring some form of management. The enormous pool of data that would be obtained through WGS NBS could serve as a powerful research tool, which could lead to a novel understanding of genetic mechanisms (Drmanac, 2012; Howard et al., 2015; Berg et al., 2017; de Wert et al., 2020). The question is, are the benefits of genomic screening such that we should shift the focus of NBS from the infant to the whole family or even the society at large. This is the question that not only scientists and clinicians but also experts from other fields and policymakers still need to answer.

If we assume that benefits for the family or society outweigh the benefits for the child, that raises another ethical issue. Testing the newborns for adult-onset diseases for the benefit of their parents would deprive them of their autonomy about decision-making regarding the results of their genetic NBS. Various international and national legal acts recognize the “right not to know” as one of the core patient’s rights. The right not to know emerged with the progress of the field of genetics. With genetic results, it is easy to imagine that a person might not want to know if he or she is going to fall ill with a late-onset disease for which there is no cure or any preventive measure. So, the right not to know was integrated in the UNESCO Universal Declaration on the Human Genome and Human Rights and also in the European Convention on Human Rights and Biomedicine (UNESCO, 1997; J Med Philos, 2000).

There is currently little known about the psychological impact of such knowledge on parents and children. Some early studies implied that there is a lingering effect of false-positive results from NBS, which makes parents of such children utilize the medical system more, but the following studies did not confirm that: Rothenberg and Sills (1968); Lipstein et al. (2009), and Tu et al. (2012). There is certainly some psychological impact on the parents, but it seems that the matter is more complex than just receiving a false-positive NBS result (Lipstein et al., 2009). With the expansion of NBS and screening for Krabbe disease, there appeared a new type of dilemma. Because of difficulties in predicting, if positive patients will develop clinical symptoms, there are children with positive results of NBS that are waiting if the disease will manifest itself. Many parents developed depression or were severely upset upon getting positive NBS results for Krabbe disease, which meant that their child might develop a devastating neurodegenerative illness. All of these symptoms were exacerbated by the fact that families were told that nothing could be done to ascertain if the disease will progress or not (Salveson, 2011; Dees and Kwon, 2013).

Many authors fear that tampering with the right not to know of neonates could lead to overdiagnosis and misdiagnosis, which could lead to mental health issues of the parents, overprotective parenting, and low self-esteem of such children. Misinterpretation and mishandling of genetic data could lead to discrimination in several areas of social life including education, employment, and health insurance (Tarini and Goldenberg, 2012; Wade et al., 2013; Frebourg, 2014; Berg et al., 2017; Murray et al., 2018). The situation of the detection of patients with a positive NBS test but an inconclusive diagnosis (CFSPID) is known in cystic fibrosis since 2005 (Cystic Fibrosis Foundation et al., 2009) and is a significant problem, since the ratio of infants with CF compared with CFSPID ranged from 1.2:1 (Poland) to 32:1 (Ireland) (Barben et al., 2017; Munck, 2020), but little is known regarding the psychological implication of such inconclusive diagnosis in families (Hayeems et al., 2017).

## Discussion

Before the NGS methodology could be implemented in NBS setting, the abovementioned obstacles and concerns have to be addressed. We need to make sure to obtain the best possible technical and methodological execution of NGS but in reasonable time regarding NBS. The choice of breadth of NGS (WGS, WES, or smaller panel) will have an impact on several aspects of NBS. Sequencing of larger parts of the genome will have as a consequence reduced capacity for multiplexing of samples, more acquired data, more complex interpretation, and higher cumulative cost. Each of the abovementioned types of approaches to NGS has its benefits and drawbacks.

Regarding present knowledge, a smaller targeted approach appears to be the better option to start the NBS. Despite slightly longer library preparation than WGS, smaller panels of genes could be sequenced faster and many more at the same time. New automated methods were already described to speed up DNA isolation and library preparation (Saavedra-Matiz et al., 2013; van Campen et al., 2019; Hendrix et al., 2020). Sequencing a smaller panel of genes would yield less data, which would require less computing power for basic data processing and subsequent analysis. Bioinformatics software pipelines for data management and variant calling should ease the load of the interpretation, but could not so far be used to automate reporting. Less data would require less disk space for storage and would be easier to integrate into existent information systems for interpretation and reporting. Such integration would enable later reinterpretation if additional knowledge about a certain gene or variant becomes available. Storage of gVCF files only would additionally reduce the size of data that required storage, so long-term data storage and data integrity could be instrumented (Berg et al., 2017; Nadeau et al., 2019; van Campen et al., 2019).

The subject of reporting of the detected genetic variants is unavoidably linked to the ethical dilemmas and psychological consequences of NBS. Knowledge about genetic idiosyncrasies of oneself or one’s child is not always good, welcomed, or easy to understand, especially when it is not unequivocal (Dees and Kwon, 2013; Howard et al., 2015; Bouvier and Giguère, 2019). So, the conditions for NGS screening should be chosen carefully to avoid unwanted consequences. Best results of NGS screening would be achieved for conditions with clear Mendelian inheritance and with good genotype–phenotype correlation, which is highly penetrant, and there is a substantial amount of knowledge on variants. To the best of our knowledge, there is yet no condition screened neonatally using NGS as a first-tier method, but cases of cascade childhood screening for familial hypercholesterolemia using NGS have been reported (Klančar et al., 2015; Knowles et al., 2017; Groselj et al., 2018). Also, some NBS centers already use NGS as second-tier confirmatory testing, and their experience could provide some of the answers (Smon et al., 2018; Lampret et al., 2020; Tangeraas et al., 2020). When we would tackle neonatal screening using NGS, we would ultimately have to decide how to report on variants found for each condition individually. Ample knowledge of incidence, prevailing genotype (like in the cases of CF and SMA) if such exists, population-specific data on gene variants, and detailed natural history for the condition would be necessary for objective decision-making regarding reporting of variants (Chien et al., 2017; Czibere et al., 2020). We propose that a unified methodology be used for the assessment of the actionability of the conditions and variants (Milko et al., 2019; Hayeems et al., 2020).

Another problem is the reporting of variants in late-onset conditions, which clashes with the right of the child not to know his or her genetic result. As much as such knowledge would benefit parents or siblings, there is not enough insight into the psychological and sociological consequences of such information on the child or parents. Too much or non-actionable information would put an unnecessary burden on parents and could cause them psychological stress; we therefore recommend that such data should not be reported until more studies are performed (Howard et al., 2015; Reinstein, 2015; Berg et al., 2017; Murray et al., 2018; de Wert et al., 2020).

We propose that the fundamental design of genetic screening remains the same as biochemical NBS, where a panel of disorders would be selected following a scrupulous selection process. The conditions included in NGS NBS should have clear actionability early in life, strong genotype–phenotype correlation, sufficient population-specific variant data, well-defined criteria for reporting, and adequate specificity and sensitivity. The reporting of the results would essentially remain the same as the results from methods currently in use in NBS. Focus would remain solely on the newborn, and such screening would possibly not need additional informed consent or counseling before testing. One option would be the use of a stepwise software decision aid that would be used to either inform parents about genetic NBS or even obtain an informed consent if needed. Nonetheless, great care should be taken on how to implement genetics into newborn screening.

In conclusion, despite several remaining obstacles, NGS will likely enter or has already entered many NBS programs in the near future. NGS has great potential to improve and expand NBS and with it our understanding of genetic mechanisms, which in turn will enable us to better diagnose conditions and offer personalized treatments. Therefore, it is necessary that we set to this task with great care and attention to ethical standards and evidence-based decision-making, to ensure a reliable and beneficial program that will continue to improve the lives of newborns and their parents.

## Author Contributions

KT and ZR conceived the project. ZR drafted the manuscript. All authors contributed to the manuscript revision, read, and approved the submitted version.

## Conflict of Interest

The authors declare that the research was conducted in the absence of any commercial or financial relationships that could be construed as a potential conflict of interest.
